# Assay methods based on proximity-enhanced reactions for detecting non-nucleic acid molecules

**DOI:** 10.3389/fbioe.2023.1188313

**Published:** 2023-06-29

**Authors:** Ye Seop Park, Sunjoo Choi, Hee Ju Jang, Tae Hyeon Yoo

**Affiliations:** ^1^ Department of Molecular Science and Technology, Ajou University, Suwon, Republic of Korea; ^2^ Department of Applied Chemistry and Biological Engineering, Ajou University, Suwon, Republic of Korea

**Keywords:** biosensor, proximity-enhanced reaction, proximity ligation assay, proximity extension assay, proximity proteolysis assay

## Abstract

Accurate and reliable detection of biological molecules such as nucleic acids, proteins, and small molecules is essential for the diagnosis and treatment of diseases. While simple homogeneous assays have been developed and are widely used for detecting nucleic acids, non-nucleic acid molecules such as proteins and small molecules are usually analyzed using methods that require time-consuming procedures and highly trained personnel. Recently, methods using proximity-enhanced reactions (PERs) have been developed for detecting non-nucleic acids. These reactions can be conducted in a homogeneous liquid phase via a single-step procedure. Herein, we review three assays based on PERs for the detection of non-nucleic acid molecules: proximity ligation assay, proximity extension assay, and proximity proteolysis assay.

## 1 Introduction

Accurately identifying and quantifying biological molecules such as proteins, small molecules, and nucleic acids plays a crucial role in the diagnosis and treatment of disease. Therefore, developing precise and reliable assays is essential for clinical medicine. Homogeneous assays have recently been developed for nucleic acids based on the specific hybridization between nucleic acid strands. This has enabled the development of simple methods for the early detection of pathogens and abnormal cells (Yan et al., 2014; Trotter et al., 2020). However, for non-nucleic molecules such as proteins and small molecules, detection methods that make use of heterogeneous assays involving solid surfaces have been the standard for several decades (Zhang et al., 2014; Cohen and Walt, 2019). Typical examples of these assays are the enzyme-linked immunosorbent assay and its modified versions, which satisfy key diagnostic features, including robustness, sensitivity, and specificity. However, the procedures involved in performing the assays involve multiple steps and require trained personnel or automated instruments. These assays can therefore be time-consuming and/or expensive. Consequently, there is an increasing demand for homogeneous assays that can detect non-nucleic acid molecules precisely without separating the target analyte from the detection molecule.

**FIGURE 1:**
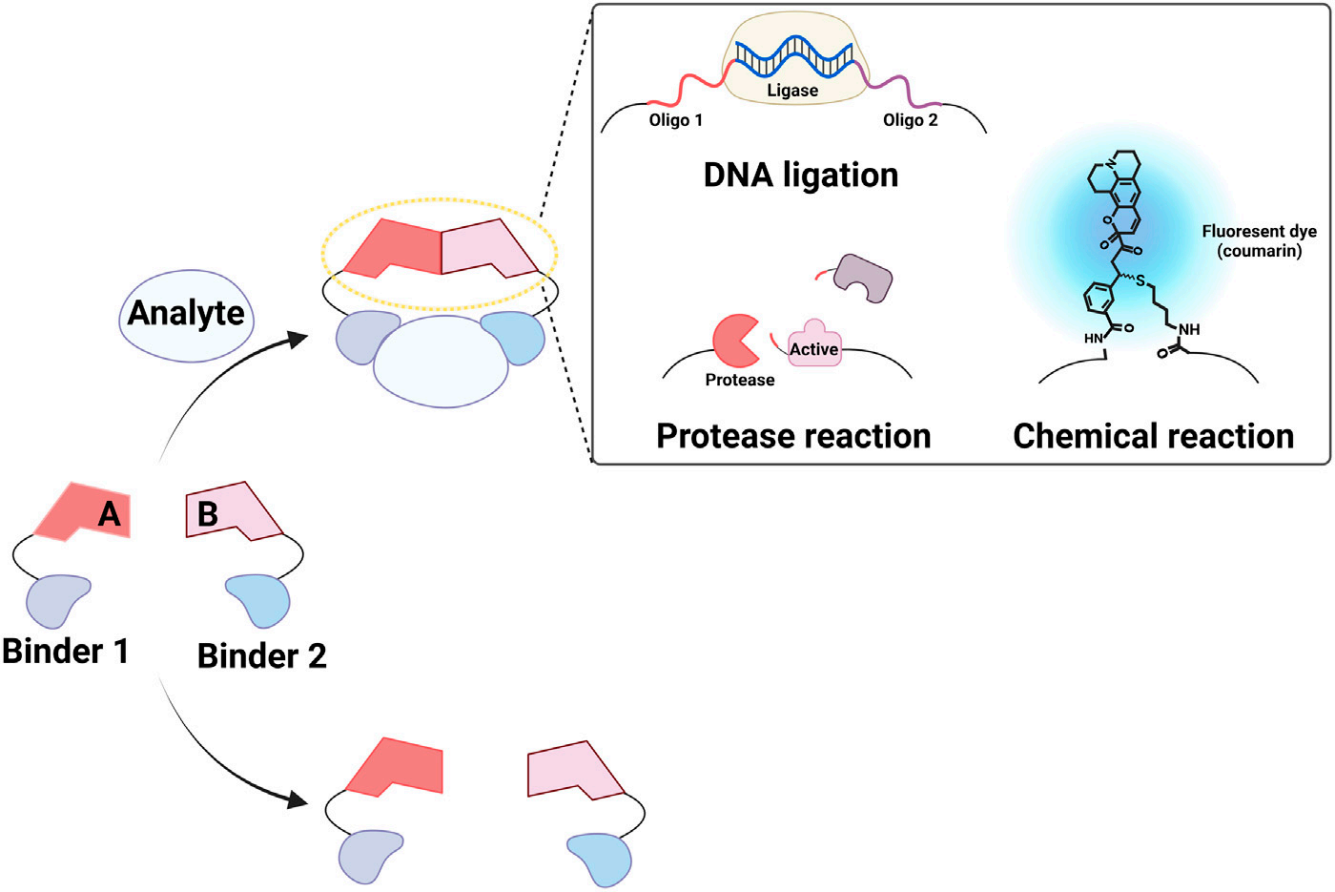
Conceptual diagram of proximity-enhanced reactions (PERs). Created with BioRender.com.

**FIGURE 2:**
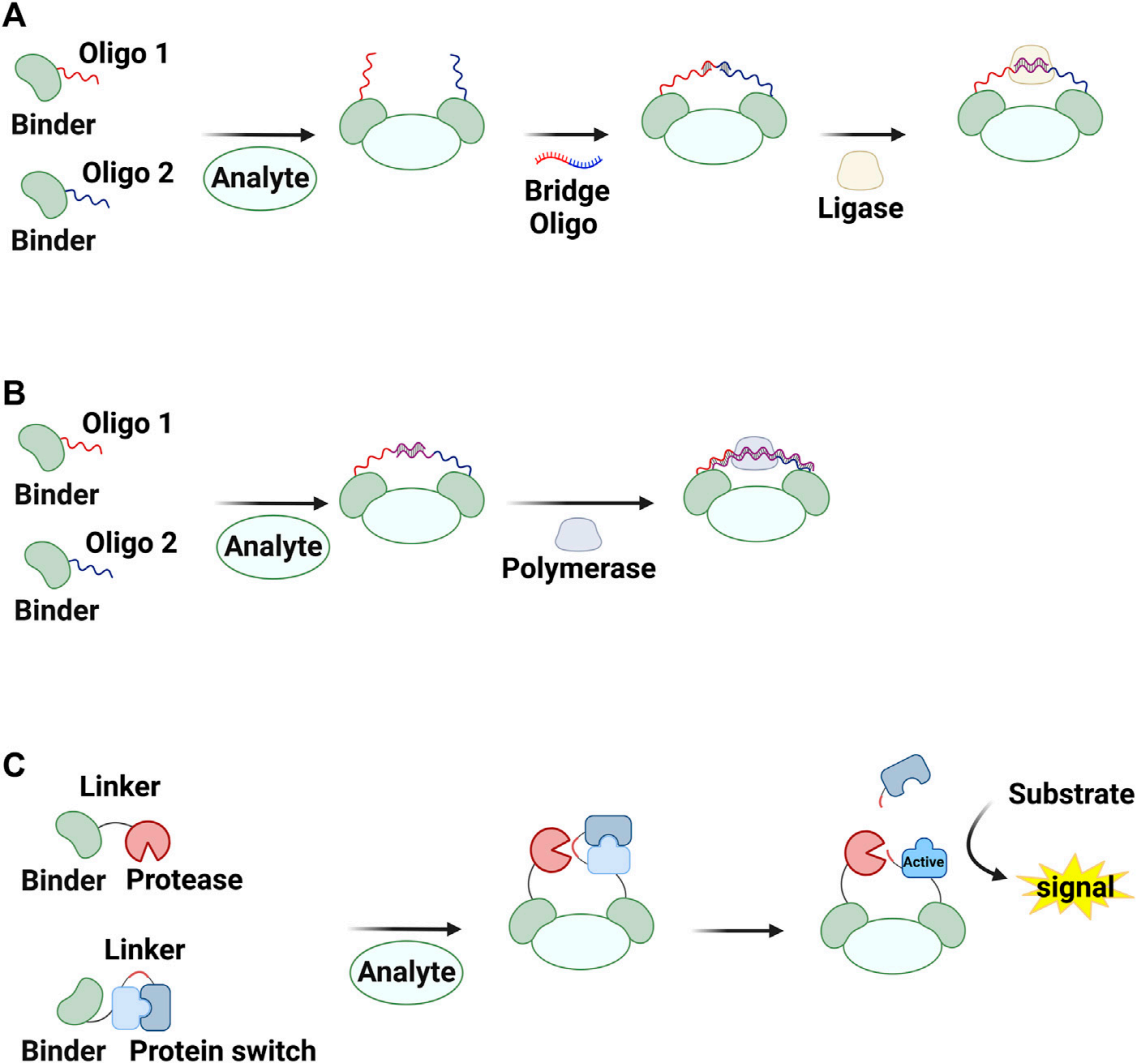
Assays based on proximity-enhanced reactions (PERs) described in this review. Shown are **(A)** a proximity ligation assay (PLA), **(B)** a proximity extension assay (PEA), and **(C)** a proximity proteolysis assay (PPA). Created with BioRender.com.

Strategies for inducing molecular assembly in the presence of target molecules have been proposed to develop homogeneous methods for detecting proteins and small molecules (Liu et al., 2016; Park and Yoo, 2018; Hwang et al., 2020). For instance, the colocalization of sensors generates a detectable signal, thereby enabling assay performance in the liquid phase with minimal background signal. Förster resonance energy transfer pairs (PJ Santangelo, 2004; Blackstock and Chen, 2014; Graham et al., 2022) and split proteins (Shekhawat and Ghosh, 2011) have both been used to monitor molecular interactions. Covalently or physically linking these molecules to two binders that target independent regions of a molecule can result in detection of the target in the homogeneous phase. When molecules linked to target binders participate in chemical or biological reactions, this reaction can be enhanced in the presence of the target via increased effective concentrations This system, called proximity-enhanced reactions (PERs), has been used to design chemical (Al Sulaiman et al., 2017; Velema and Kool, 2017; Rossetti et al., 2020) and biological reactions (Gullberg et al., 2003; Weibrecht et al., 2010; Blokzijl et al., 2014; Greenwood et al., 2015a; Greenwood et al., 2015b; Liu et al., 2016; Alam, 2018; Park and Yoo, 2018; Rossetti et al., 2020; Wang et al., 2021) for detecting various molecules, including proteins, antibodies, and nucleic acids as well as to characterize molecular interactions (Figure 1). In this review, we describe how PERs have been used to develop methods to detect non-nucleic acid molecules. We focus on three types of assays: proximity ligation assay (PLA; Figure 2A), proximity extension assay (PEA; Figure 2B), and proximity proteolysis assay (PPA; Figure 2C).

## 2 PLA

PLA, first reported 20 years ago (Fredriksson et al., 2002), involves a ligation reaction between single-stranded oligonucleotides conjugated to target-binding molecules (Figure 2A). For this reaction, a pair of binder–oligonucleotide conjugates is first placed in proximity in the presence of a target molecule. The ligation reaction, which is initiated via a bridge oligonucleotide and DNA ligase, can be enhanced in the presence of a target molecule via proximity effects. That is, the target concentration increases the yield of the ligated product. The product molecule can then be detected using various DNA amplification techniques. In this section, we first describe conjugates of target binders and oligonucleotides and then discuss methods for detecting ligated oligonucleotides.

### 2.1 Binder–oligonucleotide conjugates

Antibodies and their fragments, such as scFv and Fab, have been frequently used to prepare binder–oligonucleotide conjugates (Gullberg et al., 2004) (Table 1). Proteins based on alternative scaffolds—e.g., designed ankyrin repeat proteins (DARPins)—have also been developed as binders for targets and have been used to generate binder–oligonucleotide conjugates (Gu et al., 2013) (Table 1). Additionally, antigens have been used as binders for the detection of antibodies (Tsai et al., 2016; Tsai et al., 2018; Cortez et al., 2020; Karp et al., 2020; Cortez et al., 2022; Lind et al., 2022) (Table 1). Aptamers have also been used as binders (Fredriksson et al., 2002; Yang and Ellington, 2008; Joonyul Kim, 2010; Liu et al., 2020; Zhao et al., 2020; Marnissi et al., 2021) (Table 1); they possess a key advantage over protein binders in that binder (aptamer)–oligonucleotide molecules can be prepared as a single molecule via chemical or biological pathways (Fredriksson et al., 2002; Yang and Ellington, 2008; Joonyul Kim, 2010; Liu et al., 2020; Zhao et al., 2020; Marnissi et al., 2021).

**TABLE 1:** Summary of the applications of the three PER-based assays.

Target	Binders	Conjugation method (binders and oligonucleotides)	Detection method	Biological fluid tested	Purpose	Detection limit	References
Proximity ligation assay (PLA)							
Platelet-derived growth factor (PDGF)	Aptamers	No conjugation (synthesized)	qPCR	Yes (FCS, human CSF)	Protein detection	Zeptomole (40 × 10^−21^ mol)	Fredriksson et al. (2002)
Cytokine	Antibodies	1) thiolated oligos and SMPB-linked antibodies	qPCR	Yes (FCS)	Cytokine detection	Femtomolar (in 1 μL)	Gullberg et al. (2004)
		2) streptavidin-oligos and biotinylated antibodies					
Porcine parvovirus; *L. intracellularis*	Antibodies	streptavidin-oligos and biotinylated antibodies	qPCR	No	Pathogen detection	1 or a few copies of viral particles (in 50 μL)	Gustafsdottir et al. (2006)
						1 or a few bacteria (in 1 μL)	
Thrombin; PDGF	Aptamers	No conjugation (synthesized)	qPCR	No	Protein detection	0.8 nM (thrombin)	Yang and Ellington (2008)
						12.8 pM (PDGF)	
Thrombin	Aptamers	No conjugation (synthesized)	qPCR	No	Thrombin detection	5 amol of thrombin	Joonyul Kim (2010)
Clenbuterol (CLE); Ractopamine (RAC)	BSA	Biotinylated BSA and biotinylated oligos	qPCR	No	Detection of small molecules	0.01 ng/mL	Cheng et al. (2012)
*Clostridium difficile* Toxin	Antibodies	streptavidin-oligos and biotinylated antibodies	qPCR/digital PCR	No	Toxin detection	0.12 ng/mL	Dhillon et al. (2016)
Auto-thyroglobulin autoantibodies	Antigens	Sulfo-SMCC crosslinked antigens and thiolated oligos	qPCR	Yes (mouse serum, human patient plasma)	Detection of antibodies	Zepto- to attomoles of antibodies (in 2 μL)	Tsai et al. (2016)
Anti-HIV antibodies	Antigens	Sulfo-SMCC crosslinked antigens and thiolated oligos	qPCR	Yes (oral fluid)	Diagnosis of HIV infection	110, 880, and 550 zmol of anti-p24, anti-gp41, anti-gp160, respectively	Tsai et al. (2018)
Multiple islet autoantibodies in type 1 diabetes	Antigens	Sulfo-SMCC crosslinked antigens and thiolated oligos	qPCR	Yes (human serum)	Detection of multiple islet autoantibodies in type 1 diabetes	∼ attomoles of antibodies	Cortez et al. (2020)
							Cortez et al. (2022)
SARS-CoV-2 antibodies	Antigens	Sulfo-SMCC crosslinked antigens and thiolated oligos	qPCR	Yes (human serum)	Detection of antibodies	98.25% sensitivity	Karp et al. (2020)
COVID-19	Aptamers	Ligation of two oligos	qPCR	Yes (human serum)	COVID-19 diagnosis	37.5 pg/mL	Liu et al. (2020)
Newcastle disease virus (NDV)	Aptamers	Streptavidin-oligos and biotinylated aptamers	qPCR	Yes (nasal per cloacal swabs)	NDV diagnosis	0.58 EID_50_/mL	Marnissi et al. (2021)
O-GlcNAcylated protein	Antibodies	Sulfo-SMCC crosslinked antibodies and thiolated oligos	qPCR	Yes (serum)	Detection of protein-specific glycosylation	0.5 amol	Robinson et al. (2016)
Glycosylated protein (CD44 and E-Cadherin)	Antibodies, L-PHA lectin	streptavidin-oligos and biotinylated affinity binders	qPCR	Yes (10% chicken serum)	Detection of post-translational modifications	8 fM (CD44)	Oliveira et al. (2018)
Phosphorylated protein (p53, EGFR)						74 fM (E-Cadherin)	
						3 fM (p53)	
						6 fM (EGFR)	
O-GlcNAcylated protein	Antibodies, *Clostridium perfringen* OGA^D298N^	Sulfo-SMCC crosslinked antibodies and thiolated oligos	qPCR	No	Quantification of protein-specific glycosylation	20 pg/mL	Song et al. (2021)
SUMOylated p53	Antibodies	streptavidin-oligos and biotinylated antibodies	qPCR	No	Detection of p53 specific SUMOylation	0.69 fM	Chen and Liang (2022)
VEGF, IL-4, IL-10, IL-1α, TNFα, IL-7	Antibodies	aldehyde/hydrazine chemistry	qPCR	Yes (human plasma, chicken plasma)	Cancer marker detection		Fredriksson et al. (2007)
21 protein markers	Antibodies	aldehyde/hydrazine chemistry	qPCR	Yes (human plasma)	Profiling of putative cancer biomarkers		Fredriksson et al. (2008)
21 biomarkers	Antibodies	Probemaker PLUS and MINUS kits (Olink Biosciences)	qPCR	Yes (human plasma)	Biomarker detection for pancreatic cancer		Chang et al. (2009)
80 biomarkers	Antibodies	Lightning-Link^TM^ technology (Innova Biosciences)	qPCR	Yes (human plasma)	High-throughput protein biomarker research		Lundberg et al. (2011b)
c-myc and max	Antibodies	Sulfo-SMCC crosslinked antibodies and thiolated oligos	RCA	No	Endogenous protein-protein interactions		Soderberg et al. (2006)
Phosphorylated PDGFR	Antibodies	Antibodies and amine-modified oligos	RCA	No	Detection of phosphorylated PDGFR		Jarvius et al. (2007)
VEGFR2 and VEGFR3	Antibodies	Sulfo-SMCC crosslinked antibodies and thiolated oligos	RCA	No	Receptor-receptor interactions		Nilsson et al. (2010)
HER2	Designed ankyrin repeat proteins (DARPins)	BG (benzylguanine)-modified oligos and DARPins (cysteine)	RCA/qPCR	No	HER2 detection		Gu et al. (2013)
IL-7 receptor hetero-complex	Antibodies	Probemaker PLUS and MINUS kits (Olink Biosciences)	RCA	No	Detection of cytokine receptor dimerization		Andersen et al. (2013)
Phosphorylated tyrosine	Antibodies	Probemaker PLUS and MINUS kits (Olink Biosciences)	RCA	No	pTyr profiling		Lioudmila Elfineh (2014)
Glycosylated protein	Antibodies	thiolated oligos and SMPB linked antibodies	RCA	No	Visualization of protein-specific glycosylation		Li et al. (2017)
Methylated arginine	Antibodies	Probemaker PLUS and MINUS kits (Olink Biosciences)	RCA	No	Detection of arginine methylation		Poulard et al. (2020)
Glycosylated PD-L1	Aptamers	Ligation of two oligos	RCA	No	Imaging of glycosylated PD-L1		Fu et al. (2021)
Proximity extension assay (PEA)							
Thrombin	Aptamers	Ligation of two oligos	qPCR	No	Thrombin detection	30 pM	Di Giusto et al. (2005)
18 biomarkers	Antibodies	Lightning-Link^TM^ technology (Innova Biosciences)	qPCR	Yes (human plasma)	Detection of low-abundance proteins		Lundberg et al. (2011a)
74 biomarkers	Antibodies	Lightning-Link^TM^ technology (Innova Biosciences)	qPCR	Yes (human plasma)	Detection of serological biomarkers		Stine Buch Thorsen (2013)
Protein markers	Antibodies	Sulfo-SMCC crosslinked antibodies and thiolated oligos	qPCR	Yes (serum, plasma)	96-plex immunoassays for high throughput detection of protein markers		Assarsson et al. (2014)
Plasma proteins	Antibodies	Probemaker PLUS and MINUS kits (Olink Biosciences)	qPCR	Yes (human plasma)	Identification of candidate plasma protein biomarkers		Berggrund et al. (2019)
Streptavidin; Adenosine triphosphate (ATP)	Biotin-primers Aptamers	No conjugation (synthesized)	EXPAR	No	Detection of protein and small molecules	2.9 fM (streptavidin)	Zhang et al. (2021)
						31.3 fM (ATP)	
IL-6	Antibodies	Biotinylated oligos and antibodies with streptavidin	EXPAR	No	Protein detection	100 fM	Li et al. (2021b)
PDGF-BBThrombinPSAAFP	Aptamers, Antibodies	No conjugation (synthesized, aptamers)	EXPAR	Yes (human serum)	Protein detection	10 fM (PDGF-BB)	Hu et al. (2022)
		Biotinylated oligos and antibodies with streptavidin (antibodies)				10 fM (thrombin)	
						121 pM (PSA)	
						104 pM (AFP)	
Proximity proteolysis assay (PPA)							
Rapamycin	Rapamycin binding domains	Genetic fusion between binders and specific protease and its inhibitory domain	Fluorescence signals from activated zymogen	No	Protein detection	Below 0.5 pM	Stein and Alexandrov (2014)
ectodomain of HER2cardiac troponin Ithrombindigoxigenin (Dig)Anti-Dig antibodyAnti-hCG antibody	Aptamers, Antibodies, Digoxigenin, Hcg	Azide incorporated binders and oligos modified with dibenzocycloocyne (DBCO)	Absorbance signals from activated zymogen	Yes (mouse serum)	Protein detection	5.03 pM (ectodomain of HER2)10.51 pM (cardiac troponin I)6.82 pM (thrombin)273.9 pM (digoxigenin)78.51 pM (Anti-Dig antibody)9.83 pM (Anti-hCG antibody)	Park et al. (2021)

Various methods have been used to link protein binders with oligonucleotides. Nucleophilic groups present in proteins, such as the primary amine of lysine and the thiol of cysteine, are frequently employed to conjugate protein binders with oligonucleotides. Oligonucleotides are synthesized chemically to be capable of reacting with the nucleophiles of proteins. For example, in one study, the lysine residues of protein binders were reacted with N-hydroxysuccinimide (NHS)-modified oligonucleotides to prepare protein–oligonucleotide conjugates (Li et al., 2019). The nearly irreversible binding between biotin and streptavidin has also been used to link binders and oligonucleotides. Protein binders can be modified with biotin via the amine–NHS coupling reaction. The resulting biotinylated binders and oligonucleotides can then be assembled via streptavidin, which has four binding sites for biotin (Gustafsdottir et al., 2006; Lundberg et al., 2011a; Dhillon et al., 2016; Chen and Liang, 2022) (Table 1). In another study, the authors made use of the fast and biorthogonal reaction between tetrazine and trans-cyclooctene (TCO). Protein binders were first reacted with NHS–tetrazine and then conjugated with TCO-modified oligonucleotides (van Buggenum et al., 2016). The protein has more than one lysine residue, and its N-terminus is the primary amine. That is, conjugation strategies using amine–NHS coupling inevitably yield heterogeneous products. To overcome this limitation, a method to site-specifically introduce unnatural amino acids into proteins was used to generate binder–oligonucleotide conjugates. Acetyl phenylalanine (AcF) was incorporated into protein binders using an engineered orthogonal amber suppressor aminoacyl-tRNA synthetase/tRNA pair derived from *Methanococcus jannaschii* (Kazane et al., 2012). The acetyl group was then reacted with aminooxy-modified oligonucleotides, which produced homogeneous products.

### 2.2 Methods to detect ligated oligonucleotides

#### 2.2.1 Polymerase chain reaction (PCR)

A typical PCR produces millions to billions of copies of a specific segment of DNA. Methods of monitoring the amplification process in a real-time manner, usually via tracking fluorescent signals, are known as quantitative PCR (qPCR). They can quantify the abundance of template DNA with very high sensitivity and specificity (Fredriksson et al., 2002; Gullberg et al., 2003; Gullberg et al., 2004; Gustafsdottir et al., 2006; Fredriksson et al., 2007; Schallmeiner et al., 2007; Fredriksson et al., 2008; Yang and Ellington, 2008; Chang et al., 2009; Joonyul Kim, 2010; Masood Kamali-Moghaddam and Gholamreza Tavoosidana, 2010; Lundberg et al., 2011b; Tavoosidana et al., 2011; Cheng et al., 2012; Gu et al., 2013; Blokzijl et al., 2014; Dhillon et al., 2016; Robinson et al., 2016; Tsai et al., 2016; Jalili et al., 2018; Tsai et al., 2018; Li et al., 2019; Cortez et al., 2020; Karp et al., 2020; Liu et al., 2020; Marnissi et al., 2021; Song et al., 2021; Chen and Liang, 2022; Cortez et al., 2022; Lind et al., 2022) (Table 1). Recently, next-generation sequencing (NGS) following a short PCR has been used to detect PLA products (Darmanis et al., 2011; Nong et al., 2013).

#### 2.2.2 Rolling circle amplification (RCA)

Relying on repeated heating and cooling steps limits the application of PCR-based techniques despite their advantages. Therefore, isothermal amplification methods, such as RCA, have been used to detect the ligated nucleotides produced by PLA (Soderberg et al., 2006; Jarvius et al., 2007; Nilsson et al., 2010; Andersen et al., 2013; Gu et al., 2013; Lioudmila Elfineh, 2014; Li et al., 2017; Poulard et al., 2020; Zhao et al., 2020; Fu et al., 2021) (Table 1). For this procedure, oligonucleotides conjugated to binders are designed to yield circular DNA PLA products; these can then be used as templates for RCA reactions. The amplified products are long single-stranded DNA molecules, and in combination with fluorescent probes, this method can be useful for imaging targets (Soderberg et al., 2006; Jarvius et al., 2007; Nilsson et al., 2010; Andersen et al., 2013; Gu et al., 2013; Lioudmila Elfineh, 2014; Li et al., 2017; Poulard et al., 2020; Zhao et al., 2020; Fu et al., 2021).

### 2.3 Non-nucleic acid targets detected by PLA

The first PLA method was developed to detect platelet-derived growth factor using two aptamer binders and qPCR (Fredriksson et al., 2002). Since the development of this method, PLA methods coupled with PCR have been developed to detect various non-nucleic acid target molecules, including cytokines (Gullberg et al., 2004), pathogens (Gustafsdottir et al., 2006; Wang et al., 2021), thrombin (Yang and Ellington, 2008; Joonyul Kim, 2010), clenbuterol (Cheng et al., 2012), ractopamine (Cheng et al., 2012), toxins (Dhillon et al., 2016), antibodies (Tsai et al., 2016; Tsai et al., 2018; Cortez et al., 2020; Karp et al., 2020; Cortez et al., 2022), viruses (Liu et al., 2020; Marnissi et al., 2021), and post-translationally modified proteins (Robinson et al., 2016; Oliveira et al., 2018; Song et al., 2021; Chen and Liang, 2022) (Table 1). Multiplexed protein marker detection using PLA methods has been reported using qPCR (Fredriksson et al., 2007; Fredriksson et al., 2008; Chang et al., 2009; Lundberg et al., 2011b; Blokzijl et al., 2014) (Table 1). Moreover, PLA methods for detecting protein–protein interactions (PPIs) (Jarvius et al., 2007; Nilsson et al., 2010; Andersen et al., 2013) and post-translational modifications (PTMs) (Lioudmila Elfineh, 2014; Li et al., 2017; Poulard et al., 2020; Fu et al., 2021) via RCA have also been reported (Table 1).

#### 2.3.1 Antibodies

**FIGURE 3:**
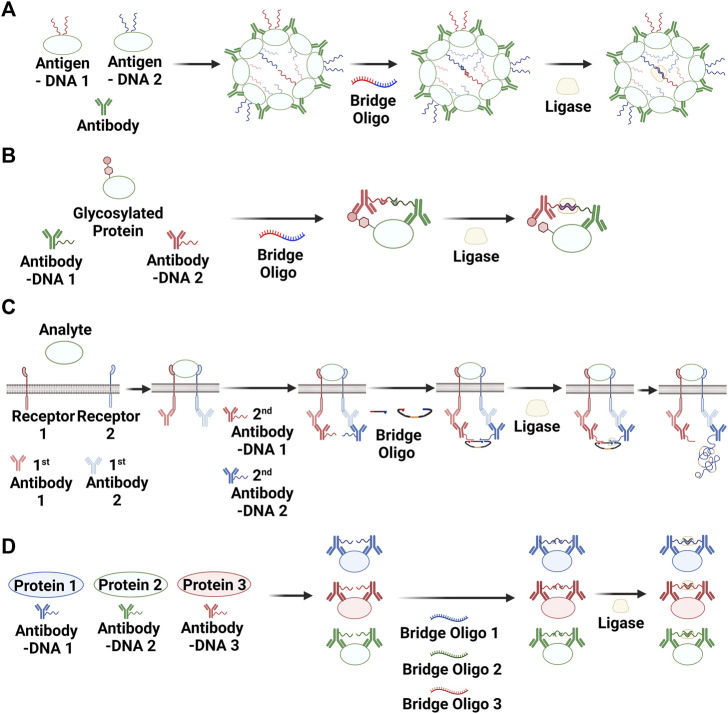
PLA methods for detecting antibodies (i.e., via agglutination-PCR) **(A)**, post-translational modifications **(B)**, protein–protein interactions **(C)**, and simultaneous multiple targeting **(D)**. Created with BioRender.com.

Antibodies are also widely used as disease biomarkers. In one case, protein antigens were conjugated with oligonucleotides to develop an antibody detection method based on PLA (Tsai et al., 2016). The presence of antibodies enhanced the ligation reaction between the oligonucleotides conjugated with antigens. The ligated products were then detected and quantified using qPCR. Because antibodies can induce the formation of aggregates in the presence of their antigen, this strategy was named “antibody detection by agglutination-PCR” (ADAP; Figure 3A). A method for detecting anti-thyroglobulin antibodies based on ADAP was found to be 1,000-fold more sensitive than an FDA-approved radioimmunoassay (Tsai et al., 2016). The ADAP method has also been used for the early diagnosis of HIV infection (Tsai et al., 2018) and for detecting multiple islet-specific autoantibodies associated with type 1 diabetes (Cortez et al., 2020). Recently, robotic systems have been developed to fully automate ADAP assays (Karp et al., 2020; Cortez et al., 2022).

#### 2.3.2 PTMs

PTMs are involved in various protein functions and are thus important for understanding diseases (Karve and Cheema, 2011; Darling and Uversky, 2018; Conibear, 2020). Immunoblotting and mass spectrometry have been widely used to identify and elucidate protein modifications (Seo and Lee, 2004; Hermann et al., 2022). Robinson et al. (2016) developed an ultrasensitive method for detecting protein glycosylation based on PLA (Figure 3B). An N-acetylgalactosamine derivative containing an azide group (N-azidoacetylgalatosaime; GalNAz) was transferred to O-N-acetylglucosamine residues on proteins via an enzymatic reaction involving glycosyl transferase. Once this step was complete, the azide group was reacted with biotin–alkyne via click chemistry. Two antibody–oligonucleotide conjugates, one for the protein of interest and the other for biotin, were used for PLA, and the ligated products were quantified using qPCR. PLA-based methods have also been reported to image glycosylated proteins *in situ* (Li et al., 2017; Fu et al., 2021). Cells were first incubated with tetra-acetylated N-azidoacetylmannosamine, and the monosaccharide as a surrogate of N-acetylmannosamine was incorporated via glycosylation into glycosylated proteins. As in the GalNAz case described above, the azide group was reacted with biotin–alkyne via the click chemistry. RCA instead of PCR was used to image the glycosylated proteins following the ligation reaction. Lectins (Oliveira et al., 2018) and an engineered β-D-N-acetylglucosaminidase mutant (Song et al., 2021) have also been used as binders for glycosylated proteins.

#### 2.3.3 PPIs

The ability to observe transient PPIs is an important tool for understanding key cellular events and their consequences. In particular, the *in situ* visualization of PPIs can provide additional information for studying dynamic biological processes. Methods consisting of proximity ligation reaction and imaging of the product with RCA and fluorescent probes have been applied to detect numerous PPIs, including c-Myc and Max (Soderberg et al., 2006), VEGFR2 and VEFGR3 (Nilsson et al., 2010), and the IL-7 receptor hetero-complex (Andersen et al., 2013) (Figure 3C).

#### 2.3.4 Multiple targets

The discovery of biomarkers and their applications for diagnosis or treatment have played a central role in clinical medicine (Blokzijl et al., 2014; Davis et al., 2020). Given the complexity of biological processes, detecting one biomarker within a sample does not usually provide enough information for understanding the disease state (Chang et al., 2009; Zemans et al., 2017). Thus, there is an ongoing need for methods capable of analyzing multiple biomarkers from a small amount of a single sample. A multiplexed protein detection method based on PLA was first reported in 2007 (Fredriksson et al., 2007), and this strategy has been used to identify biomarkers for various cancers (Figure 3D). Furthermore, NGS after a short PCR has been used to examine the composition of a mixture of ligated oligonucleotides (Darmanis et al., 2011; Nong et al., 2013), which enables the analysis of many biomarkers from one sample.

## 3 PEA

**FIGURE 4:**
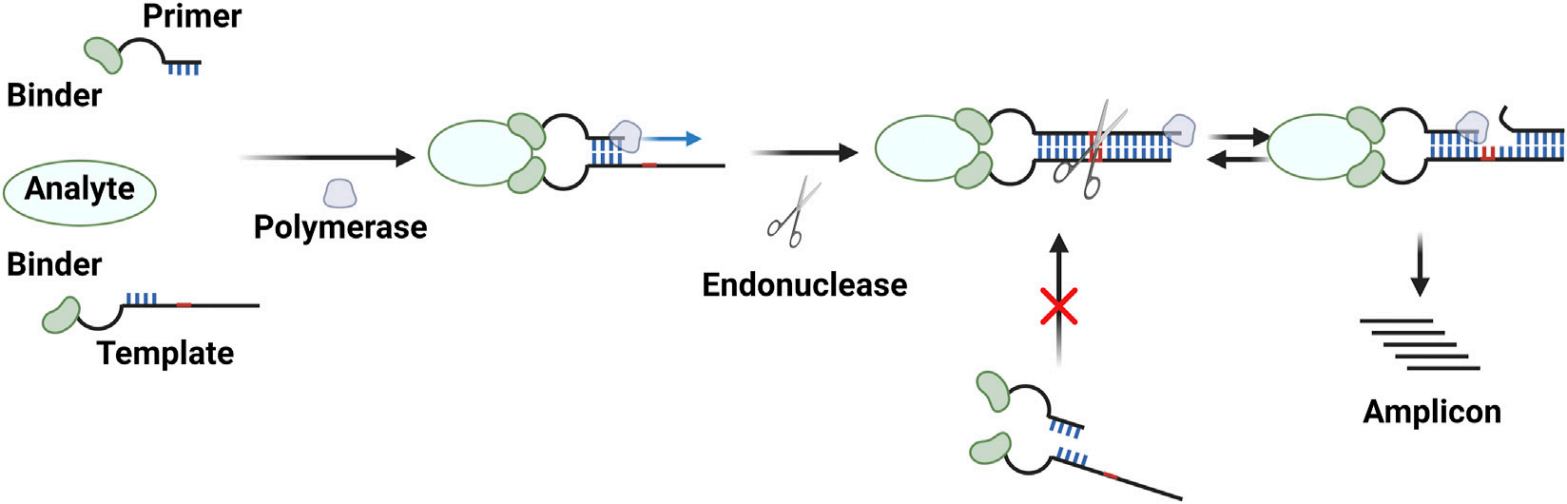
Diagram showing the PEA method with an exponential amplification reaction (EXPAR). Created with BioRender.com.

PEAs also rely on reaction enhancement based on a pair of binder–oligonucleotide conjugates. In this procedure, two single-stranded oligonucleotides conjugated to binders hybridize with each other in the presence of a target molecule via the proximity effect, and DNA polymerase then generates double-stranded DNAs (Figure 2B). The resulting amplicons can be analyzed using PCR or isothermal amplification methods (Di Giusto et al., 2005; Lundberg et al., 2011a; Lundberg et al., 2011b; Stine Buch Thorsen, 2013; Assarsson et al., 2014; Berggrund et al., 2019; Li et al., 2021b; Zhang et al., 2021; Hu et al., 2022) (Table 1). PEA has the same advantages as PLA, including reaction homogeneity, high sensitivity, high specificity, and low sample consumption. Moreover, PEA has been found to be less sensitive to reaction conditions. For example, T4 DNA polymerase used in a typical PEA protocol performed well in blood plasma, which is different from DNA ligases for PLA (Lundberg et al., 2011a; Lundberg et al., 2011b). Lundberg et al. (2011a) reported a PEA method for analyses of human blood. This method enabled the sensitive and specific detection of low-abundance proteins from human blood plasma. In combination with qPCR or NGS, this method has been used for multiplex analyses of human proteomes in high throughput ways (Lundberg et al., 2011a; Stine Buch Thorsen, 2013; Assarsson et al., 2014; Berggrund et al., 2019; Wik et al., 2021). The assay itself has been commercialized by Olink (Uppsala, Sweden). While RCA is typically used as an isothermal amplification method for PLA, another strategy, termed the exponential amplification reaction (EXPAR) has been used for PEA (Figure 4) (Li et al., 2021b; Zhang et al., 2021; Hu et al., 2022). Strand-displacing DNA polymerases such as phi29 DNA polymerase and Bst DNA polymerase (large fragment) are used instead of T4 DNA polymerase to generate double-stranded DNAs. One strand of the product is cleaved by nicking endonucleases, and then the strand-displacing DNA polymerases synthesize DNA strands starting from the nicked site, yielding ssDNA amplicons. These amplicons can be detected in various ways, including fluorescence signals coming from G-quadruplexes complexed with thioflavin T (Zhang et al., 2021), molecular beacons (Hu et al., 2022), and Cas12a activation (Li et al., 2021b).

## 4 PPA

**FIGURE 5:**
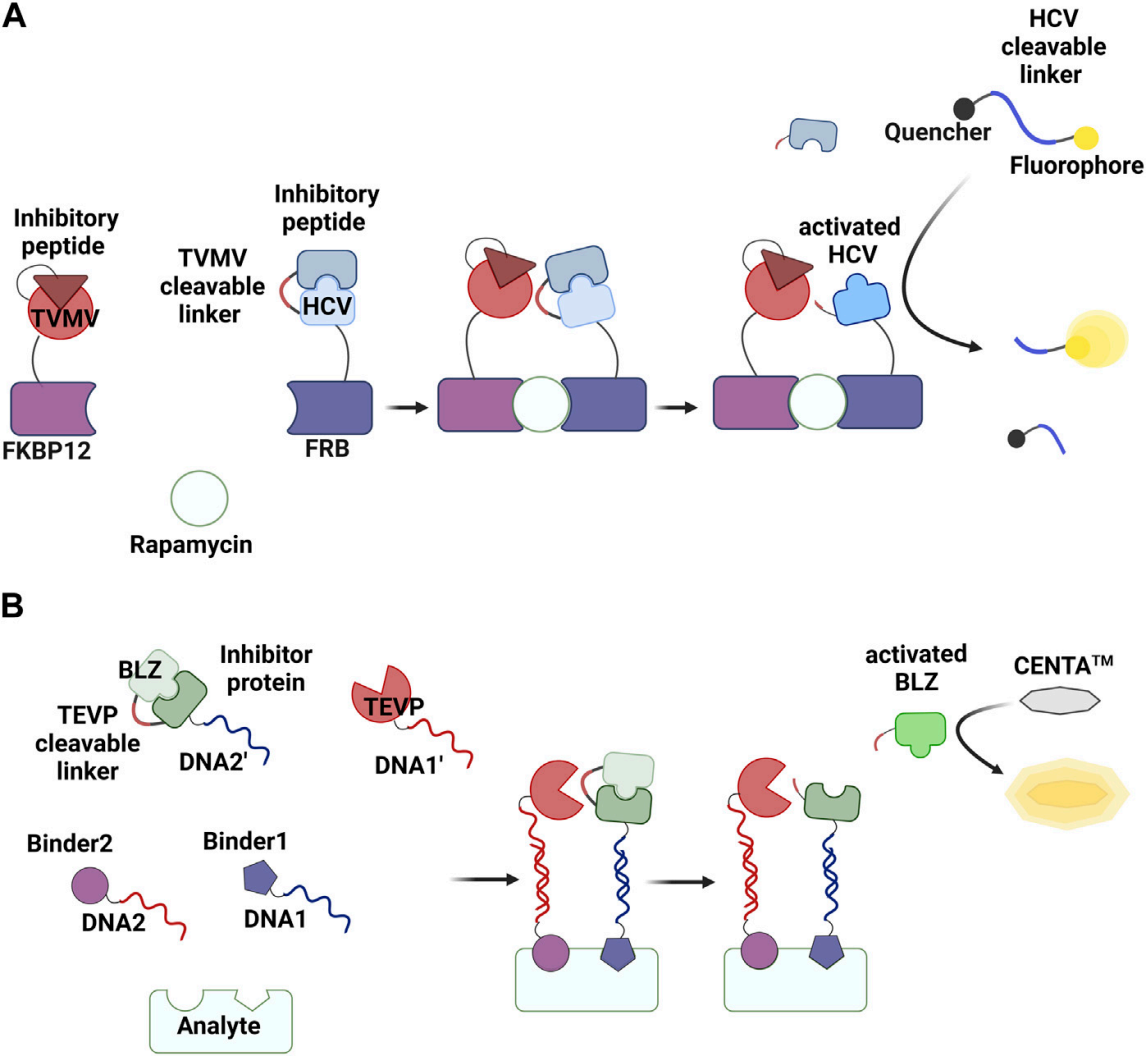
PPA methods reported by Stein and Alexandrov (Stein and Alexandrov, 2014) **(A)** and by Park et al. (Park et al., 2021) **(B)**. Created with BioRender.com.

The concept of enhancing reaction rates via proximity can be generalized from ligase- and polymerase-mediated reactions to many other types of chemical and enzymatic reactions. Several research groups, including us, have recently reported PER-based assay methods. One of these, termed PPA, has been successfully developed into methods for detecting non-nucleic acid targets (Table 1). A protease and its substrate (i.e., zymogen) are linked to target binders. The proteolysis reaction is enhanced in the presence of the target, and the activated zymogen generates a detectable signal (Figure 2C). The first example of a PPA method used to detect a non-nucleic acid molecule was reported by Stein and Alexandrov (Stein and Alexandrov, 2014) (Figure 5A). Zymogen was designed as a hepatitis C virus NS3 serine protease (HCV) was connected to its inhibitory peptide via a flexible linker. The linker includes an amino acid sequence that is cleavable by a nuclear inclusion a (NIa) protease from tobacco vein mottling virus (TVMV). TVMV was also fused to its inhibitory peptide to decrease its activity, and this can reduce background signal in the absence of a target of interest. The authors developed a homogeneous method to detect rapamycin by fusing two engineered proteases to rapamycin-binding proteins (the FRB and FKBP12 domains). A proteolysis reaction by TVMV was found to be enhanced in the presence of rapamycin, and the activated HCV generated a fluorescence signal by hydrolyzing a quenched substrate.

Park et al. (2021) developed a PPA system that can be used for detecting various molecules (Figure 5B). In this method, a protease (tobacco, etch virus protease; TEVP) and an engineered β-lactamase zymogen (BLZ) (Kim et al., 2014) that can be activated by TEVP are linked to target binders via specific hybridization between complementary DNAs. Conjugation between proteins and oligonucleotide was achieved via a strain-promoted click reaction between azide and cyclooctyne (Park and Yoo, 2018). An azide-containing unnatural amino acid (4-azido-phenylalanine; AzF) was site-specifically introduced into these proteins using an orthogonal pair of tRNA and an aminoacyl-tRNA synthetase engineered to specifically incorporate AzF into the amber codon (Park et al., 2021; Park et al., 2022). The single-stranded DNAs were modified with dibenzocycloocyne. Various molecules, including antibodies, proteins, aptamers, and small molecules, have been used as target binders, and different strategies were employed to conjugate target binders with oligonucleotides. This assay can be conducted in a one-pot format by incubating four conjugates (TEVP-DNA1’, BLZ-DNA2’, Binder1-DNA1, and Binder2-DNA2) and a chromogenic substrate for β-lactamase (CENTA™) with samples, after which a change in absorbance is measured. Homogeneous assay methods have successfully been developed for detecting various analytes, including the ectodomain of human epidermal growth factor receptor-2, cardiac troponin I, thrombin, digoxigenin (Dig), and anti-Dig antibody, at subnanomolar concentrations using a one-step procedure and color signal.

## 5 Conclusion

One strategy to develop methods for detecting biomarkers is via inducing molecular assembly in the presence of a target, which in turn generates a detectable signal. When molecules participating in chemical or biological reactions are parts of the molecular assembly, the reaction between the molecules can be enhanced by their increased effective concentrations. This concept, known as PER, has been used to develop methods for detecting and quantifying non-nucleic acid molecules in many studies. Herein, we described three types of assays based on PERs: PLA, PEA, and PPA. While assays for detecting nucleic acids are usually conducted in a homogenous liquid, the detection of non-nucleic acid molecules often depends on methods involving multiple steps (ELISA) or suffers from limited sensitivities (LFA) (Table 2). PER-based approaches enable the development of homogenous assays for detecting targets such as proteins, small molecules, molecular interactions, and nucleic acids. These simple and sensitive assays can identify and quantify the content of many biological molecules, which is crucial for the diagnosis and treatment of diseases.

**TABLE 2:** Features of assays for detecting non-nucleic molecules.

	Advantages	Disadvantages/Limitations	Remarks
ELISA (Enzyme-linked immunosorbent assay)	*High robustness, sensitivity, and specificity	*Multiple steps for binding with targets and removing non-specific interactions (i.e., heterogeneous)	*Sample-to-answer assay time is < 4 h
		*Trained personnel or automated instruments are needed	*Limit of detection is ∼10^–15^ M
		*Binders may not be available	*Can detect the presence of small quantities of a substrate, either antigen or antibody
			*Reproducible
LFA (Lateral flow assay)	*Simple and fast procedure (i.e., easy to use)	*Qualitative or semi-quantitative signal with a limited sensitivity	*Sample-to-answer assay time is < 15 min
	* No laboratory equipment and no extensive training required	*Binders may not be available	*Limit of detection is ∼10^–6^ M
	*Lightweight and portable	*False-positive results from prolonged interaction between signaling unit and capture molecules	
	*Long storage stability		
	*Cheapest (e.g., human chorionic gonadotropin (pregnancy) LFAs, are <$1 per test)		
PLA (Proximity ligation assay)	*High specificity and sensitivity, fast, high throughput, and versatile	*Methods (i.e., PCR or RCA) are needed for detecting ligated oligonucleotides	
	*Some assays can be done in the homogeneous phase	*Binders may not be available	
	*Low sample consumption	*Conjugation between binders and oligonucleotides may be needed	
	*Localized detection		
PEA (Proximity extension assay)	*High specificity and sensitivity, fast, high throughput, and versatile	*Methods (i.e., PCR or EXPAR) are needed for detecting extended oligonucleotides	
	*Some assays can be done in the homogeneous phase	*Binders may not be available	
	*Low sample consumption	*Conjugation between binders and oligonucleotides may be needed	
	*Less sensitive to reaction conditions		
	*Localized detection		
PPA (Proximity proteolysis assay)	*Homogeneous reaction (i.e., one-pot reaction)	*Binders may not be available	
	*Activated zymogen in proximity can amplify the signals	*Conjugation between binders and oligonucleotides may be needed	

The strategy used to design binder–oligonucleotide conjugates is modular, and various binders—including ones that were previously investigated for targets—can be used to develop assay methods based on PERs. At present, antibodies are the most used binders for PERs; which sometimes involve complicated processes to link antibodies and oligonucleotides (Tables 1, 2). On the contrary, aptamers have several advantages over antibodies, including their small size, high stability, and production via chemical synthesis (Thiviyanathan and Gorenstein, 2012; Li et al., 2021a). Moreover, binder (aptamer)–oligonucleotide molecules can be synthesized as one-strand oligonucleotides, and this process is much simpler and cheaper than many of the methods used to conjugate protein binders and chemically modified oligonucleotides. Currently, the number of available aptamers is much smaller than that of antibodies. However, technological improvement of instruments and advances in automation are expected to accelerate the discovery of target-specific aptamers (Michael Famulok and blind, 2000; Shaban and Kim, 2021).

Olink (Uppsala, Sweden) has successfully launched products based on its PEA technology, and one of them (Olink^®^ Explore 3072) coupled with NGS readouts enables ∼3000 protein assays in a high-throughput way. However, point-of-care tests (POCTs) based on PERs have not yet been widely developed, probably because of the detection methods of PER-based assays. For example, fluorescent signals are most commonly used to measure the products of PLAs and PEAs, and the detection of these signals requires complex instruments for quantitative analysis. Several POCTs for detecting nucleic acids have been very recently developed, and these were mainly related to the SARS-CoV-2 pandemic (Islam and Iqbal, 2020; Kang et al., 2022; Ye et al., 2022). Incorporating the newly development methods would be one approach to developing POCTs based on PLAs and PEAs. The PPA (Figure 4B) used the β-lactamase zymogen as a reporter to produce an absorbance signal. This can be quantified by a relatively simple instrument, such as a smartphone (Bergua et al., 2022). Thus, this method has potential for the development of POCTs to detect non-nucleic acid targets. Moreover, the strategy used to design the β-lactamase zymogen can be applied to engineer other reporter enzymes (Inoue et al., 2010; Stein and Alexandrov, 2015) into zymogens, and various such PPAs are expected to be developed in the future.
